# Pediatric Seizure Team-based Learning

**DOI:** 10.21980/J8MD22

**Published:** 2020-07-15

**Authors:** Sara Paradise Dimeo, Gabriel Sudario, Supriya Sharma, Lilly Bellman, Anjalee Gallion, Katherine Andreeff, Megan Boysen-Osborn

**Affiliations:** * University of South Carolina, Greenville, Prisma Health-Upstate Department of Emergency Medicine, Greenville, SC; ^ University of California, Irvine, Department of Emergency Medicine, Orange, CA; † Harbor UCLA Medical Center, Department of Emergency Medicine, Torrance, CA; ** CHOC Children’s, Division of Pediatric Neurology, Orange, CA; ^^ CHOC Children’s, Pediatric Hospitalist; University of California, Irvine, Department of Pediatrics, Orange, CA

## Abstract

**Audience:**

The target audiences for this team-based learning are emergency medicine and emergency medicine-pediatric resident physicians.

**Introduction/Background:**

Pediatric seizure is a common presenting complaint in the emergency department. It is said that over 470,000 children have a diagnosed seizure disorder^1^ and 2%–5% of children aged 6 months to five years will have a febrile seizure at some point during childhood.^2^ While there are many published educational materials related to pediatric seizure, they are simulation-based, and/or isolated to management of one underlying diagnosis.^3^,^4^,^5^,^6^ Therefore, this team-based learning uses four cases to provide an understanding of the possible causes of seizure in children, as well as the management, workup, and disposition for emergency medicine residents in training.

**Educational Objectives:**

By the end of this TBL session, learners should be able to:

1. Define features of simple versus complex febrile seizure
2. Discuss which patients with seizure may require further diagnostic workup
3. Summarize a discharge discussion for a patient with simple febrile seizures
4. Identify a differential diagnosis for pediatric patients presenting with seizure
5. Define features of status epilepticus
6. Review an algorithm for the pharmacologic management of status epilepticus
7. Indicate medication dosing and routes of various benzodiazepine treatments
8. Obtain a thorough history in an infant patient with seizures to recognize hyponatremia due to improperly prepared formula
9. Choose the appropriate treatment for a patient with a hyponatremic seizure
10. Describe the anatomy of a ventriculoperitoneal (VP) shunt
11. Relate a differential diagnosis of VP shunt malfunction
12. Compare and contrast the neuroimaging options for a patient with a VP shunt

**Educational Methods:**

This team-based learning is a classic TBL because it contains learner responsible content (LRC), an individual readiness assessment test (iRAT), a multiple-choice group RAT (gRAT) with immediate feedback assessment technique (IF/AT), and a group application exercise (GAE).

**Research Methods:**

We received formative feedback through conversations with learners afterwards, who stated they enjoyed the activity and felt it was highly useful for their learning; in addition, instructors discussed after the session and made changes accordingly.

**Results:**

We collected verbal feedback from instructors and learners after the session. Learners and instructors felt that it was very successful with limited modifications, in particular, the need for more time to complete the activity. Therefore, we suggest a 90 minute rather than 60-minute timeframe to adequately cover all material.

**Discussion:**

Pediatric seizure is a common complaint in the emergency department. It can be a difficult subject for the emergency medicine resident to master based on the variety of presentations. Indeed, the cause, management, and disposition may vary greatly; the etiology may range from benign to life-threatening, sometimes requiring minimal and at other times an extensive workup, with an ultimate disposition of either discharge home or admission to a pediatric intensive care unit. Therefore, team-based learning is well-suited to work through some of the complexities of such cases, and we found this educational session to be highly effective.

**Topics:**

Pediatric seizure, simple febrile seizure, complex febrile seizure, status epilepticus, hyponatremic seizure, ventriculoperitoneal (VP) shunt, team-based learning.

## USER GUIDE

[Table t1-jetem-5-3-t1]

**Table t1-jetem-5-3-t1:** 

List of Resources:	
Abstract	1
User Guide	3
Learner Materials	6
iRAT	6
gRAT	8
GAE	11
Instructor Materials	20
RAT Key	21
GAE Key	25

**Learner Audience:**

Interns, junior residents, senior residents

**Time Required for Implementation:**

Instructor Preparation: 60–90 minutes

Learner Responsible Content: 0–60 minutes

In Class Time: 90 minutes

**Recommended Number of Learners per Instructor:**

While this cTBL can be taught with only one instructor, ideally, there will be additional facilitators to interact with small groups during the group application exercise.

Therefore, it is recommended to have 1 instructor per every 10–20 learners.

**Topics:**

Pediatric seizure, simple febrile seizure, complex febrile seizure, status epilepticus, hyponatremic seizure, ventriculoperitoneal (VP) shunt, team-based learning.

**Objectives:**

By the end of this TBL session, learners should be able to:

1. Define features of simple versus complex febrile seizure
2. Discuss which patients with seizure may require further diagnostic workup
3. Summarize a discharge discussion for a patient with simple febrile seizures
4. Identify a differential diagnosis for pediatric patient presenting with seizure
5. Define features of status epilepticus
6. Review an algorithm for the pharmacologic management of status epilepticus
7. Indicate medication dosing and routes of various benzodiazepine treatments
8. Obtain a thorough history in an infant patient with seizures to recognize hyponatremia due to improperly prepared formula
9. Choose the appropriate treatment for a patient with a hyponatremic seizure
10. Describe the anatomy of a ventriculoperitoneal (VP) shunt
11. Relate a differential diagnosis of VP shunt malfunction
12. Compare and contrast the neuroimaging options for a patient with a VP shunt

### Linked objectives and methods

Pediatric seizure is a common complaint in the emergency department. It can be a difficult subject for the emergency medicine resident to master based on the variety of presentations. Indeed, the cause, management, and disposition may vary greatly; the etiology may range from benign to life-threatening, sometimes requiring minimal and at other times an extensive workup, with an ultimate disposition of either discharge home or admission to a pediatric intensive care unit. Therefore, team-based learning is well-suited to work through some of the complexities of such cases, including simple versus complex febrile seizures (objectives 1, 2, and 3), a pediatric patient in status epilepticus (objectives 4, 5, 6, and 7), the treatment of an infant with a hyponatremic seizure who ultimately is receiving watered-down infant formula (objectives 8 and 9), and unique situations which may predispose a patient to seizure, such as a ventriculoperitoneal shunt (objectives 10, 11, 12). Building from simple definition to more complex decisions, including a treatment algorithm and disposition, these cases provide emergency medicine resident physicians with dedicated practice on managing these cases.

### Recommended pre-reading for instructor

- Richer L, Mikrogianakis A, Helman A. Episode 73: Emergency Management of Pediatric Seizures. Emergency Medicine Cases. https://emergencymedicinecases.com/emergency-management-of-pediatric-seizures/. Published November 2015. Accessed May 30, 2019.
- Clinical Report of the American Academy of Pediatrics. Febrile seizures: clinical practice guideline for the long-term management of the child with simple febrile seizures. *Pediatrics*. 2008;121(6):1281–1286.
- Grenga P, Bodkin R, Rotoli J, Silberstein H. Complications of CSF Shunts: ED Presentations, Evaluation and Management. emDocs. http://www.emdocs.net/complications-csf-shunts-ed-presentations-evaluation-management/. Published November 2017. Accessed May 31, 2019.

### Learner Responsible Content (LRC)

Prior to the session, learners should review the following materials:

- Richer, L, Mikrogianakis, A, Helman A. Episode 73: Emergency Management of Pediatric Seizures. Emergency Medicine Cases. https://emergencymedicinecases.com/emergency-management-of-pediatric-seizures/. Published November 2015. Accessed May 30, 2019.
- Grenga P, Bodkin R, Rotoli J, Silberstein H. Complications of CSF Shunts: ED Presentations, Evaluation and Management. emDocs. http://www.emdocs.net/complications-csf-shunts-ed-presentations-evaluation-management/. Published November 2017. Accessed May 31, 2019.

### Results and tips for successful implementation

Our team-based learning was conducted in the format suggested below. While no formal assessment was collected, we received formative feedback through conversations with learners afterwards, who stated they enjoyed the activity and felt it was useful for their learning. Based on the instructor discussions after the activity, it was felt to be successful with limited modifications such as the need for more time; therefore, we suggest a 90 minute rather than 60-minute timeframe to adequately cover all material. Additionally, it is highly recommended that you have each group start with a different case, thus ensuring all cases are covered and each group participates in group discussion.

*Instructions for implementation:*

Prior to the session, the instructor should prepare materials:

- One individual readiness assessment test (iRAT) per learner
- One group readiness assessment test (gRAT) per 3–5 learners (one per group)
- One group application exercise (GAE) per 3–5 learners (one per group)
- Copies of all materials, including the keys, for each instructor

You will need approximately 90 minutes to conduct the session. We suggest the following timeline:

1. Introduce session (1 minute)
2. The instructor hands out the iRAT to all learners and have them complete it individually (5–10 minutes)
3. The instructor assigns learners into groups of 3–5. Ideally, a mix of level of learners is best (ie, interns, juniors, and seniors). Groups complete the gRAT (5–10 minutes). *See gRAT section for how to prepare a gRAT.*
4. The instructor briefly reviews answers for the gRAT (5–10 minutes); a suggested format is to call on groups one at a time to give their answer. Note: this is NOT a time for in-depth discussion of questions, but rather just pre-assessment review of answers. Assure learners that material will be covered in greater detail during cases.
5. Groups complete the GAE (30 minutes); again, we suggest having all groups complete all cases whenever possible; however, you may assign some groups to start at case 1, some groups to start at case 2, some groups to start at case 3, etc., so that all cases are covered when time is called.
6. Review answers from the GAE (30 minutes); a suggested format is to call on groups one at a time to answer each question. Note that the instructor materials provide enhanced answers in case of questions, and not all material will need to be covered.
7. Hand out post-test learning sheet.

## Figures and Tables

**Figure:**
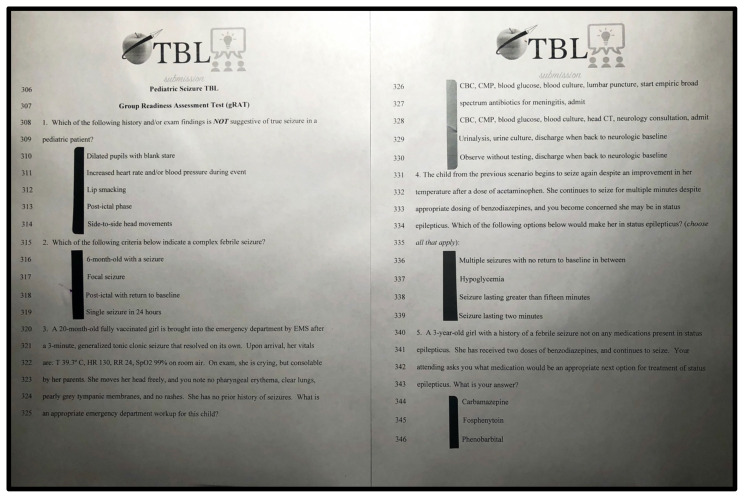


**Table t2-jetem-5-3-t1:** 

Medication	Dose (mg/kg)	Route Options	Maximum Doses
1.			
2.			
3.			

**Table t3-jetem-5-3-t1:** 

Your labs result with the following:	
Complete Blood Count:	Basic Metabolic Panel:
White blood cells 11,200/mm^3^	Sodium 110 mEq/L
Hemoglobin 16.2 g/dL	Potassium 5.6 mEq/L
Hematocrit 46.5%	Chloride 85 mEq/L
Platelets 253 ×10^3^/mm^3^	Bicarbonate 16 mEq/L
	Blood urea nitrogen 12.1 mEq/L
	Creatinine 0.6 mEq/

**Figure f2-jetem-5-3-t1:**
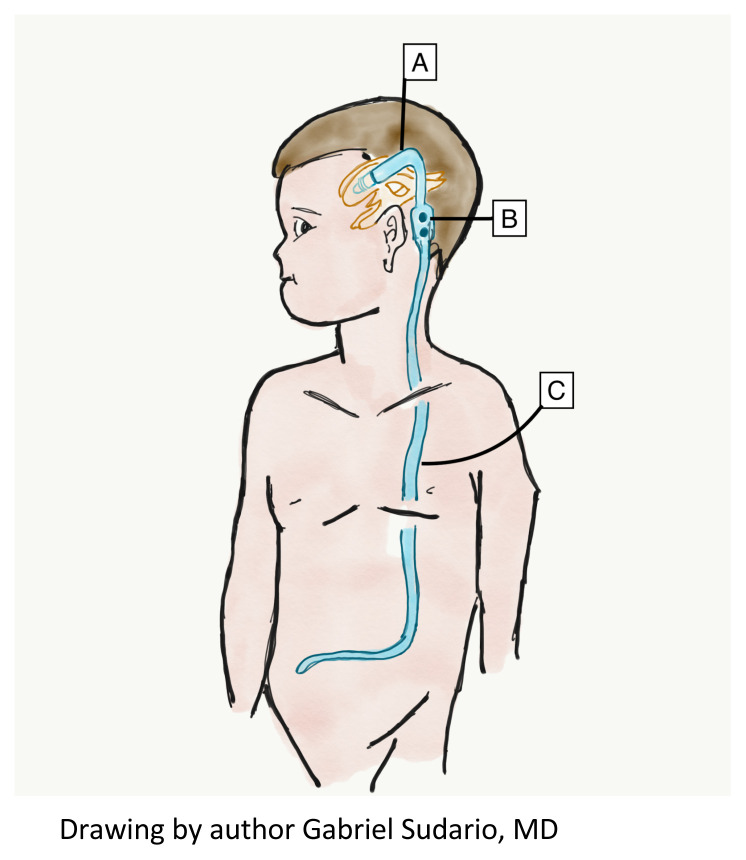
Drawing by author Gabriel Sudario, MD

**Table t4-jetem-5-3-t1:** 

Fever Source Exam	Neurologic Exam
**HEAD** If fontanelle present, check for bulging (Also check for signs of trauma such as cephalohematoma) **EENT** Ears for otitis media (also for hemotympanum) Throat for vesicles, erythema Mouth for herpetic lesions Nose for nasal congestion Throat for vesicles, erythema **NECK** Does child move head freely? Any neck stiffness or signs of meningeal irritation? **RESPIRATORY** Assess work of breathing Auscultate for stridor, rales, wheezing **CARDIOVASCULAR** Tachycardia (appropriate for fever?) Capillary refill <3 seconds? **ABDOMEN** Any suprapubic tenderness **SKIN** Any rashes? Look at palms, soles for hand/foot mouth	**MENTAL STATUS** “Alert, verbal, pain, unresponsive” (AVPU) scale (Note: Glasgow coma score is a trauma score and is not appropriate in post-seizure evaluation) Observe interaction with parents/providers - Playful/interactive? - Fussiness? Is child appropriately consolable? - Are there signs of true irritability? Ie, child is paradoxically fussier with attempted calming measures by parents. **SPEECH** Developmentally normal language for age (at least with parents) **CRANIAL NERVES** Pupillary response Tracking object or person with eyes Facial symmetry Developmentally appropriate tasks (eg, follow a toy with eyes for extraocular movements) **MOTOR** Evaluate muscle tone Developmentally appropriate tasks like reaching for toys with both hands, giving high fives **SENSORY** Observe/evaluate grossly **COORDINATION** Note from motor tasks - Observe movements, reaching for toys, reaching for parents, walking, truncal stability while sitting

**Table t5-jetem-5-3-t1:** 

Medication	Dose (mg/kg)	Route Options	Maximum Doses
1.			
2.			
3.			

**Table t6-jetem-5-3-t1:** 

Initial treatment options for pediatric seizure^12^			
Medication	Dose (mg/kg)	Route Options	Maximum Dose
1. Lorazepam	0.1 mg/kg	Intramuscular (IM), Intravenous (IV)	4 mg
2. Midazolam	0.2 mg/kg	IM, IV, buccal, intranasal	10 mg (max 5 mg per nares for intranasal route)
3. Diazepam	2–5 years old: 0.5 mg/kg	Rectal (PR)	20 mg
3. Diazepam (continued)	6–11 years old: 0.3 mg/kg >12 years old: 0.2 mg/kg	Rectal (PR)	20 mg

**Table t7-jetem-5-3-t1:** 

Differential Diagnosis	Historical Questions	Physical Examination Findings
1. Infection	Fevers? Lethargy? Sick contacts?	- Check rectal temperature - Perform full physical exam to look for any possible infectious sources (ie, fontanelles, lungs, abdomen, TMs (tympanic membranes), throat, nose, skin).
2. Metabolic disturbance	*Genetic abnormality*: Poor weight gain, abnormal facial features, suggestive family history *Hyponatremia*: How is formula being mixed? Intake and output?	
3. Ingestions/toxins	Any exposures to medications/toxins?	- Check pupils - Check reflexes - Check muscle tone
4. Trauma (accidental, non-accidental)	Any trauma?	- Check for signs of trauma, including bruising

**Table t8-jetem-5-3-t1:** 

Your labs result with the following:	
Complete Blood Count:	Basic Metabolic Panel:
White blood cells 11,200/mm^3^	Sodium 110 mEq/L
Hemoglobin 16.2 g/dL	Potassium 5.6 mEq/L
Hematocrit 46.5%	Chloride 85 mEq/L
Platelets 253 ×10^3^/mm^3^	Bicarbonate 16 mEq/L
	Blood urea nitrogen 12.1 mEq/L
	Creatinine 0.6 mEq/

**Figure f3-jetem-5-3-t1:**
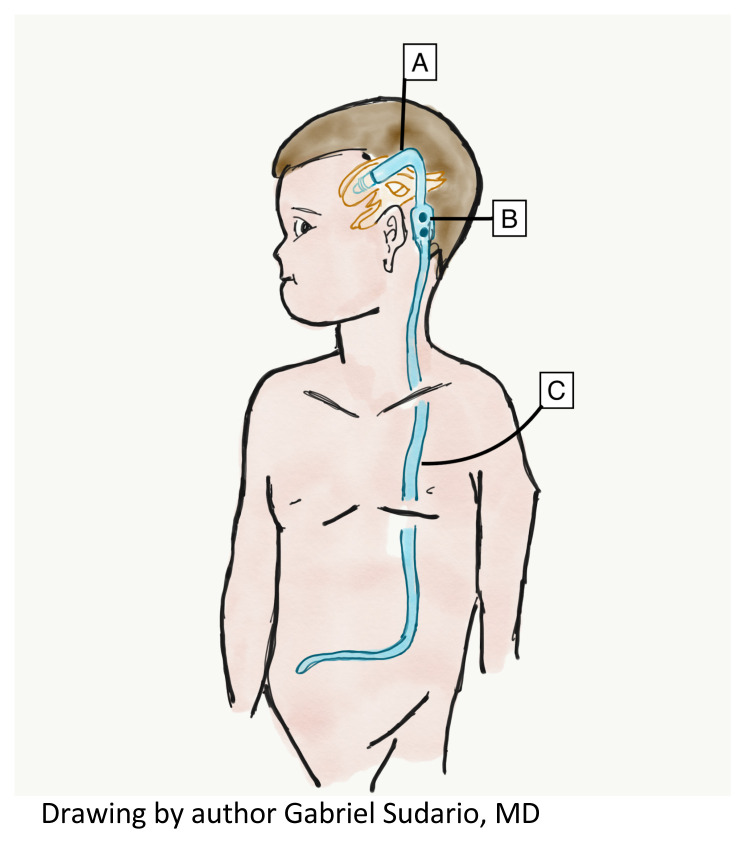
Drawing by author Gabriel Sudario, MD

## LEARNER MATERIALS

### Pediatric Seizure TBL: individual Readiness Assessment Test (iRAT)

1. All of the following history and/or exam findings are suggestive of a true seizure in a pediatric patient *EXCEPT*
  - Dilated pupils with blank stare
  - Increased heart rate and/or blood pressure during event
  - Lip smacking
  - Post-ictal phase
  - Side-to-side head movements
2. Which of the following presentations would be considered a complex febrile seizure?
  - 6-month-old with a seizure
  - Focal seizure
  - Post-ictal with return to baseline
  - Single seizure in 24 hours
3. A 20-month-old fully vaccinated girl is brought into the emergency department by EMS after a 3-minute, generalized tonic-clonic seizure that resolved on its own. Upon arrival, her vitals are: T 39.3o C, HR 130, RR 24, SpO2 99% on room air. On exam, she is crying, but consolable by her parents. She moves her head freely, and you note no pharyngeal erythema, clear lungs, pearly grey tympanic membranes, and no rashes. She has no prior history of seizures. What is an appropriate emergency department workup for this child?
  - CBC, CMP, blood glucose, blood culture, lumbar puncture, start empiric broad spectrum antibiotics for meningitis, admit
  - CBC, CMP, blood glucose, blood culture, head CT, neurology consultation, admit
  - Urinalysis, urine culture, discharge when back to neurologic baseline
  - Observe without testing, discharge when back to neurologic baseline
4. The child from the previous scenario begins to seize again despite an improvement in her temperature after a dose of acetaminophen. She continues to seize for multiple minutes despite appropriate dosing of benzodiazepines, and you become concerned she may be in status epilepticus. The child would be considered to be in status epilepticus based on which of the following? (*choose all that apply*):
  - Multiple seizures with no return to baseline in between
  - Hypoglycemia
  - Seizure lasting greater than fifteen minutes
  - Seizure lasting two minutes
5. A 3-year-old girl with a history of a febrile seizure not on any medications presents in status epilepticus. She has received two doses of benzodiazepines and continues to seize. Your attending asks you what medication would be an appropriate next option for treatment of status epilepticus. What is your answer?
  - Carbamazepine
  - Fosphenytoin
  - Phenobarbital
  - Valproic acid
6. A 3-month-old infant presents to the ED with her mother after a seizure. After taking a careful history, the mother states she has been watering down infant formula to save money. The patient begins to seize again. What is your immediate treatment?
  - Normal saline bolus
  - 3% hypertonic saline
  - 25% hypertonic saline
  - Rapid acting benzodiazepine
7. Which of the following would be an indication for neuroimaging in a pediatric patient with a seizure?
  - 5-year-old vaccinated child with a first-time febrile seizure
  - 8-year-old with a ventriculoperitoneal shunt in place
  - 10-year-old type I diabetic patient with a hypoglycemic seizure
  - 15-year-old with a history of epilepsy presenting with generalized seizure

### Pediatric Seizure TBL: group Readiness Assessment Test (gRAT)

Ideally, to make a gRAT using the immediate feedback assessment technique (IF/AT) you may purchase silver “scratch off stickers” (available at amazon.com) and place the stickers over the index letters as demonstrated in the example below. If you do not want to create a gRAT in this way, you can simply use the iRAT as a gRAT and either give a blank iRAT copy to each group or have them work together to decide on their final answers.

1. All of the following history and/or exam findings are suggestive of a true seizure in a pediatric patient *EXCEPT*
  a. Dilated pupils with blank stare
  b. Increased heart rate and/or blood pressure during event
  c. Lip smacking
  d. Post-ictal phase
  ♥ Side-to-side head movements
2. Which of the following presentations would be considered a complex febrile seizure?
  a. 6-month-old with a seizure
  ♥ b. Focal seizure
  c. Post-ictal with return to baseline
  d. Single seizure in 24 hours
3. A 20-month-old fully vaccinated girl is brought into the emergency department by EMS after a 3-minute, generalized tonic-clonic seizure that resolved on its own. Upon arrival, her vitals are: T 39.3o C, HR 130, RR 24, SpO2 99% on room air. On exam, she is crying, but consolable by her parents. She moves her head freely, and you note no pharyngeal erythema, clear lungs, pearly grey tympanic membranes, and no rashes. She has no prior history of seizures. What is an appropriate emergency department workup for this child?
  a. CBC, CMP, blood glucose, blood culture, lumbar puncture, start empiric broad spectrum antibiotics for meningitis, admit
  b. CBC, CMP, blood glucose, blood culture, head CT, neurology consultation, admit
  ♥ c. Urinalysis, urine culture, discharge when back to neurologic baseline
  d. Observe without testing, discharge when back to neurologic baseline
4. The child from the previous scenario begins to seize again despite an improvement in her temperature after a dose of acetaminophen. She continues to seize for multiple minutes despite appropriate dosing of benzodiazepines, and you become concerned she may be in status epilepticus. The child would be considered to be in status epilepticus based on which of the following? (*choose all that apply*):
  ♥ a. Multiple seizures with no return to baseline in between
  b. Hypoglycemia
  ♥ c. Seizure lasting greater than fifteen minutes
  d. Seizure lasting two minutes
5. A 3-year-old girl with a history of a febrile seizure not on any medications presents in status epilepticus. She has received two doses of benzodiazepines and continues to seize. Your attending asks you what medication would be an appropriate next option for treatment of status epilepticus. What is your answer?
  a. Carbamazepine
  ♥ b. Fosphenytoin
  c. Phenobarbital
  d. Valproic acid
6. A 3-month-old infant presents to the ED with her mother after a seizure. After taking a careful history, the mother states she has been watering down infant formula to save money. The patient begins to seize again. What is your immediate treatment?
  a. Normal saline bolus
  ♥ b. 3% hypertonic saline
  c. 25% hypertonic saline
  d. Rapid acting benzodiazepine
7. Which of the following would be an indication for neuroimaging in a pediatric patient with a seizure?
  a. 5-year-old vaccinated child with a first-time febrile seizure
  ♥ b. 8-year-old with a ventriculoperitoneal shunt in place
  c. 10-year-old type I diabetic patient with a hypoglycemic seizure
  d. 15-year-old with a history of epilepsy presenting with generalized seizure

### Pediatric Seizure TBL: Group Application Exercise (GAE)

#### Case 1

You’re getting an ambulance call! The nurse alerts you that you’ll be receiving a 16-month-old boy, purple on the Broselow. Per preliminary report from medics, he had a seizure in the field and currently is postictal with a Glasgow Coma Scale of 8. Estimated time of arrival is 10 minutes.

1. How do you prepare for this child’s arrival? What specific equipment or treatments should you have on standby?

The patient is here…. on arrival, EMS briefs you that the child had a generalized, tonic-clonic seizure that resolved just as they arrived. Since the seizure ended, the boy has been sleepy, but awakens to mother’s voice by turning to her. He is moving all four extremities. Blood glucose was checked and is 190. An intravenous line was not established prior to arrival.

On initial history, parents state he had been well today, but “maybe a bit clingier than usual.” He had just woken from his nap and while in his mother’s arms, his eyes rolled back and he started shaking “all over.” As he was shaking, she put a pen in his mouth “to stop him from swallowing his tongue.” They estimate it lasted 10 minutes (however, on further EMS prompting, it was witnessed to be only 2 minutes). On exam, the patient is protecting his airway, has equal breath sounds bilaterally and capillary refill is <3 sec. His tympanic membranes are clear. His tongue shows a superficial bite but no gross lacerations or active bleeding. His abdomen is soft. He has no rashes.

Vitals: Rectal temperature 39.1 degrees Celsius, heart rate 165, blood pressure 90/60, respiratory rate 24, pulse oximetry 99% on room air

2. What is your differential diagnosis for a febrile pediatric patient with a seizure? (List at least five).
  1)
  2)
  3)
  4)
  5)
3. Other than the history that has already been obtained, what other history questions do you want to ask that might help you narrow your differential diagnosis?
4. What are important elements of the physical exam when evaluating this boy? How do you do a neurologic exam on a child of this age?
5. What are the criteria for simple febrile seizure? Does he meet criteria?
6. What is your workup and management for this patient while in the ED?
7. Your patient has returned to his neurologic baseline and has no identifiable source of infection. At this time, you feel he can be safely discharged home. How do you counsel parents regarding:
  - Risk for further seizures?
  - Risk for epilepsy?
  - Need for antiepileptic drug therapy?
  - Fever control at home?
  - Seizure safety?
8. When do children with febrile seizures potentially need a neurology consultation or referral?

#### Case 2

An otherwise healthy 2-year-old immunized child presents to the emergency department via EMS with a complaint of seizure. Per mother, they estimate that ten minutes prior to arrival, she became unresponsive and began “shaking.” EMS reports they administered 5 mg intramuscular midazolam with no response about 5 minutes ago and have been providing supplemental oxygen. Per mother, the child is fully vaccinated. She has a history of a one-time febrile seizure as an infant and has had no issues since that time. Mother denies any recent travel. She is in daycare and lives at home with her father and 1 older sibling.

On initial exam, the child is having tonic-clonic movements with her head and eyes deviated to the right.

Vitals: Temperature 38.3 degrees Celsius, heart rate 130, blood pressure 80/60, pulse oximetry 93% on nasal cannula at 2 liters/minute

1. What are your initial priorities in management (non-medication management)?
2. The patient’s blood sugar returns at 110 mg/dl. The patient continues to seize. You would like to give another dose of benzodiazepine -- what are your options? Anything else you would like to give at this time? Note: The child is a yellow on Broselow tape, and her mother states she weighs approximately 12 kg.
  List at least 3 options, with appropriate dosages and routes.[Table t2-jetem-5-3-t1]
3. Despite your initial interventions, the child continues to seize; it has been about 20 minutes since the onset. Your nurse was able to obtain intravenous access. What is your concern now? Does this change your concern? What is your next option for the treatment of this seizure? Include dosing/route of administration.
4. Would you like to obtain any further workup on the patient at this time?
5. Despite your 3rd treatment, the patient continues to seize for an additional 10 minutes. She is becoming more hypoxic, and has a capillary refill of 3 seconds. What intervention(s) would you take at this time?
6. After your interventions, the patient’s pupils are now midline, 3 mm and reactive, and the child is not moving. What are your next steps in workup, and the ultimate disposition?

#### Case 3

A 2-week-old newborn female is brought to the emergency room by her parents after having a witnessed generalized seizure at home which resolved after 2 minutes. Per their report, she was born at term via spontaneous vaginal delivery with no complications; mother was otherwise healthy and received full prenatal care. On your initial examination, she is no longer seizing but appears to have poor tone and a weak cry. Patient weighs approximately 3.5 kg.

1. What is your differential diagnosis for this newborn infant? What questions can you ask family and examinations can you perform to develop your prioritized differential diagnosis?

Upon questioning, you note that the family has recently become homeless and are struggling financially. The mother states that she has been mixing 1 scoop of infant formula to 6 ounces of water to make the formula last longer until her next Women, Infants, and Children’s (WIC) appointment.

2. What is your concern at this time? What is your initial management and workup?[Table t3-jetem-5-3-t1]
3. Based on these laboratory studies, what is your suspected diagnosis? How is this defined?
4. The patient suddenly begins seizing again. What is your immediate management (including medication dose and route)?
5. After your intervention, the patient’s seizure stops and her vitals are stable. What is your ultimate disposition? What is the feared complication of correcting for this problem too rapidly?

#### Case 4

A 5-year-old boy with a history of congenital hydrocephalus with a VP shunt presents to the emergency department with a reported seizure at home. Per the patient’s mother, he has complained of headache for the last 4 hours and was witnessed to have a generalized seizure lasting three minutes that self-resolved. On exam he appears sleepy, but is moving all extremities and following some commands from his mother. On ocular exam he appears to have dilated pupils with an upward gaze palsy.

Vitals: Temperature 37.8 degrees Celsius, heart rate 120, blood pressure 100/55, respiratory rate 26, oxygen saturation 98% on room air

1. Based on the history above, what is your differential diagnosis for this patient? What is your initial workup and/or management?
2. Please name each component of a standard VP shunt shown in figure 1. What is the function of the VP shunt? How does it work?
  A)
  B)
  C)[Fig f2-jetem-5-3-t1]
3. On re-evaluation, the patient continues to be drowsy, with no change in clinical status. Your labs return as unremarkable. The CT scan shows acute hydrocephalus; therefore, you are concerned that the shunt may be malfunctioning. What is your next step in management? What are the possible causes of shunt malfunction? List 4 below:
  1)
  2)
  3)
  4)
4. The consultant is recommending admission to their service and requesting an MRI. Is it safe for patients with VP shunts to have an MRI?

Answer keys to all exercises with explanations, are on the following pages.

Learners: Please do not proceed.

### Pediatric Seizure TBL: Readiness Assessment Test Key (RAT Key)

1. All of the following history and/or exam findings are suggestive of a true seizure in a pediatric patient *EXCEPT*
  a. Dilated pupils with blank stare
  b. Increased heart rate and/or blood pressure during event
  c. Lip smacking
  d. Post-ictal phase
  ♥ Side-to-side head movements
    **Answer: (E);** Side-to-side head, arm, or leg movements are characteristic of non-epileptiform seizures. Dilated pupils with blank stare, increased heart rate or blood pressure during event, lip smacking, and a post-ictal phase are all characteristics of true seizure activity in pediatric patients.
2. Which of the following presentations would be considered a complex febrile seizure?
  a. 6-month-old with a seizure
  ♥ b. Focal seizure
  c. Post-ictal with return to baseline
  d. Single seizure in 24 hours
    **Answer: (B);** A complex febrile seizure includes one that is focal, rather than generalized. Or use this question: Features of a *simple* febrile seizure include:
  Brief, lasting <15 minutes
  Age 6 months to 5 years, making choice (A) above incorrect
  Return to baseline after seizure, making choice (C) above incorrect
  Single seizure in 24 hours rather than recurrent seizures, making choice (D) above incorrect
3. A 20-month-old fully vaccinated girl is brought into the emergency department by EMS after a 3-minute, generalized tonic-clonic seizure that resolved on its own. Upon arrival, her vitals are: T 39.3° C, HR 130, RR 24, SpO2 99% on room air. On exam, she is crying, but consolable by her parents. She moves her head freely, and you note no pharyngeal erythema, clear lungs, pearly grey tympanic membranes, and no rashes. She has no prior history of seizures. What is an appropriate emergency department workup for this child?
  a. CBC, CMP, blood glucose, blood culture, lumbar puncture, start empiric broad spectrum antibiotics for meningitis, admit
  b. CBC, CMP, blood glucose, blood culture, head CT, neurology consultation, admit
  ♥ c. Urinalysis, urine culture, discharge when back to neurologic baseline
  d. Observe without testing, discharge when back to neurologic baseline
  **Answer: (C); Urinalysis, urine culture, discharge when back to neurologic baseline.** The child in this clinical vignette meets criteria for a simple febrile seizure (Brief lasting <15 minutes, generalized, and one episode in 24 hours). In this case, the urinalysis and culture are an appropriate workup to evaluate fever in a 20-month-old girl with no clear source on exam.^7^ These children then can be observed in the ED until return to neurologic baseline, making choice (C) correct. Per the AAP guidelines regarding simple febrile seizures, laboratory and imaging workup in the setting of febrile seizure should serve the purpose of evaluating source of fever, not solely to evaluate for cause of seizure,^8^ thus making choices (A) and (B) incorrect as there are no clinical signs of meningitis or indications for neuroimaging.
4. The child from the previous scenario begins to seize again despite an improvement in her temperature after a dose of acetaminophen. She continues to seize for multiple minutes despite appropriate dosing of benzodiazepines, and you become concerned she may be in status epilepticus. The child would be considered to be in status epilepticus based on which of the following? (*choose all that apply*):
  ♥ a. Multiple seizures with no return to baseline in between
  b. Hypoglycemia
  ♥ c. Seizure lasting greater than fifteen minutes
  d. Seizure lasting two minutes
  **Answer: (A), (C);** Based on expert consensus and best available research, status epilepticus is defined as an abnormal tonic-clonic seizure of *at least* five minutes (the time when treatment should be initiated), with the potential for long-term neuronal injury starting at around thirty minutes of seizure activity.^9^ Additionally, there may be a lack of return to baseline after a seizure (which may suggest recurrent seizures), thus making choices (A) and (C) the correct answers. While uncorrected hypoglycemia may be a cause of status epilepticus, it is not included in the definition. A seizure lasting about 2 minutes is not long enough to consider treatment, making choice (D) incorrect.
5. A 3-year-old girl with a history of a febrile seizure not on any medications presents in status epilepticus. She has received two doses of benzodiazepines and continues to seize. Your attending asks you what medication would be an appropriate next option for treatment of status epilepticus. What is your answer?
  a. Carbamazepine
  ♥ b. Fosphenytoin
  c. Phenobarbital
  d. Valproic acid
  **Answer: (B);** In a patient >2 months of age who has received 1–2 doses of weight-based dosing of benzodiazepine medication, it is recommended to use a loading dose of a longer acting anti-epileptic drug such as fosphenytoin, making choice (B) correct. Fosphenytoin is a water-soluble pro-drug of phenytoin and has a number of advantages. Unlike phenytoin, it may be given both intravenously or intramuscularly and at a rapid rate of infusion without caustic tissue damage.^10^ Phenobarbital has a higher risk of hypotension and/or respiratory depression than fosphenytoin, making choice (C) incorrect. Valproic acid and carbamazepine are both maintenance anti-epileptic therapies; however, they should not be infused for treatment of status epilepticus, making choices (A) and (D) incorrect.
6. A 3-month-old infant presents to the ED with her mother after a seizure. After taking a careful history, the mother states she has been watering down infant formula to save money. The patient begins to seize again. What is your immediate treatment?
  a. Normal saline bolus
  ♥ b. 3% hypertonic saline
  c. 25% hypertonic saline
  d. Rapid acting benzodiazepine
  **Answer: (B);** In this patient who is seizing due to watered-down infant formula, the most likely cause of the seizure is hyponatremia. Therefore, 3% hypertonic saline should be immediately administered at a dose of 3–5 ml/kg,^11^ as benzodiazepines (D) will not be effective in this case. Normal saline, or choice (A), does not have a high enough sodium concentration to guarantee seizure cessation. 25% hypertonic saline is a very high dose, and may overcorrect the sodium, putting a patient at risk for osmotic demyelination syndrome, or central pontine myelinolysis.
7. Which of the following would be an indication for neuroimaging in a pediatric patient with a seizure?
  a. 5-year-old vaccinated child with a first-time febrile seizure
  ♥ b. 8-year-old with a ventriculoperitoneal shunt in place
  c. 10-year-old type I diabetic patient with a hypoglycemic seizure
  d. 15-year-old with a history of epilepsy presenting with generalized seizure
  **Answer: (B);** For non-febrile seizures, neuroimaging is indicated for patients with ventriculoperitoneal shunts, making choice (B) correct. Patients with a febrile seizure do not require neuroimaging as the yield is low, making choice (A) incorrect. Patients who are found to be hypoglycemic do not require neuroimaging should their mental status and neurologic exam improve after administration of glucose, making choice (C) incorrect. Additionally, patients with known seizure disorder presenting with a generalized seizure, or choice (D), do not require neuroimaging unless otherwise recommended by a consultant.

### Pediatric Seizure TBL: Group Application Exercise (GAE) Key

#### Case 1

You’re getting an ambulance call! The nurse alerts you that you’ll be receiving a 16-month-old boy, purple on the Broselow. Per preliminary report from medics, he had a seizure in the field and currently is postictal with a Glasgow Coma Scale of 8. Estimated time of arrival is 10 minutes.

1. How do you prepare for this child’s arrival? What specific equipment or treatments should you have on standby?
  - Make a resuscitation-ready room available (like a trauma bay)
  - Assign specific roles to team members
  - Prepare airway equipment: Per Broselow, 3.5mm ETT, Miller 1 blade
  - Suction equipment, pediatric bag valve mask
  - Prepare seizure medications
    ○ Midazolam 0.2 mg/kg (max 10mg)
      ▪ Intramuscular, intranasal (with mucosal atomizer), buccal administration if no IV access^12^

The patient is here…. on arrival, EMS briefs you that the child had a generalized, tonic-clonic seizure that resolved just as they arrived. Since the seizure ended, the boy has been sleepy, but awakens to mother’s voice by turning to her. He is moving all four extremities. Blood glucose was checked and is 190. An intravenous line was not established prior to arrival.

On initial history, parents state he had been well today, but “maybe a bit clingier than usual.” He had just woken from his nap and while in his mother’s arms, his eyes rolled back and he started shaking “all over.” As he was shaking, she put a pen in his mouth “to stop him from swallowing his tongue.” They estimate it lasted 10 minutes (however, on further EMS prompting, it was witnessed to be only 2 minutes). On exam, the patient is protecting his airway, has equal breath sounds bilaterally and capillary refill is <3 sec. His tympanic membranes are clear. His tongue shows a superficial bite but no gross lacerations or active bleeding. His abdomen is soft. He has no rashes.

Vitals: Rectal temperature 39.1 degrees Celsius, heart rate 165, blood pressure 90/60, respiratory rate 24, pulse oximetry 99% on room air

2. What is your differential diagnosis for a febrile pediatric patient with a seizure? (List at least five).
  - **Febrile seizure:** Febrile seizures are fairly common, occurring in ~2%–5% of children.^2^ They are defined as a fever with a temperature of at least 38 degrees Celsius (100.4 degrees Fahrenheit).^13^ A seizure may be the first presenting sign of a fever, and can be triggered by a number of pathologies, including acute otitis media, gastroenteritis, upper respiratory infections, and roseola (human herpes virus 6). Vaccinations such as measles-mumps-rubella or diphtheria-tetanus-pertussis have also been associated with febrile seizures. There may be an underlying sodium channel mutation and a family history of febrile seizure, though in about 50% of patients, no trigger is found.^14^ There are simple and complex febrile seizures (to be discussed further below).
  - **Intracranial infection such as meningitis or encephalitis:** This patient has been previously healthy with fever <24 hours, and has no concerning physical exam findings for meningitis/encephalitis. Per AAP guidelines, one would consider a lumbar puncture in cases where a child was truly irritable, has meningismus or focal neurologic deficits on exam, prolonged altered mental status, or a prolonged seizure (>15–30 minutes). Per expert opinion and case reports, you may also consider in some cases if the child is <12 months old, or the patient was under vaccinated with *Haemophilus influenzae* type B (Hib) or pneumococcal vaccine (PCV), or there was a recent antibiotic course of two weeks (theoretical partially treated meningitis)^8^.
  - **Head trauma (accidental or non-accidental):** Always consider this in a patient with a seizure. However, this child has a fever, no reported trauma, no other bruising or skin findings, and the family seems appropriately concerned with a history that corroborates with the story.
  - **Toxic ingestion:** Always consider ingestion on differential diagnosis of seizure. Multiple drugs can cause pediatric seizures, including antidepressants (such as Bupropion), anticholinergics/antihistamines, sympathomimetics, isoniazid, and others^15^.
  - **Metabolic abnormality:** Hypoglycemia, hyponatremia; this child has a normal point of care glucose, and no other significant risk factors for hyponatremia
  - **Others:** Genetic disorders, brain malformations, malignancy
  - **Seizure mimics**: Consider breath holding spell, especially in patients 6–18 months of age (however, this usually has a preceding emotional trigger and patient will return to baseline immediately). Consider syncope with myoclonic jerking, but less likely as patient had no LOC (loss of consciousness) before onset. Consider pseudoseizure but doubt in an infant, since this is very uncommon in children less than 6–7 years of age.
3. Other than the history that has already been obtained, what other history questions do you want to ask that might help you narrow your differential diagnosis?
  - History of present illness: Describe exactly what happened and the events leading up to the seizure.
    ○ What did the seizure look like? What parts of the body were moving (one side versus both sides)? What were their eyes/head doing?
    ○ Any tongue biting or urinary incontinence?
    ○ First event or any prior history of seizures- febrile or otherwise?
    ○ Any preceding trauma?
    ○ How long did it last? How long was the patient “shaking”? (Note: parents may include post-ictal somnolence and incorrectly estimate time)
    ○ Any associated symptoms (ie, upper respiratory symptoms, pulling at ears, vomiting, diarrhea/GI illness, rashes, sick contacts)?
  - Past medical history:
    ○ Birth history
    ○ Vaccination status; in particular any recent vaccinations
    ○ Past history of seizures (febrile or non-febrile)
    ○ Developmental history
  - Family History: Any family history of febrile seizures or other seizure disorder in first-degree relatives?
  - Exposures: any medications at home? Could the child have gotten into something toxic?
4. What are important elements of the physical exam when evaluating this boy? How do you do a neurologic exam on a child of this age?
  - Physical examination should be thorough, with an emphasis on two main goals:
    1. Evaluating for possible source of the fever, and

Performing an age and developmentally appropriate neurologic exam

This chart is a non-exhaustive summary of some physical exam elements to consider:[Table t4-jetem-5-3-t1]

5. What are the criteria for simple febrile seizure? Does he meet criteria?
  - Brief (<15 minutes)
  - Generalized, tonic-clonic (ie, non-focal)
  - No more than one episode in 24 hours
  - Age 6–60 months (five years old)
  - Fever
  - Developmentally normal
6. What is your workup and management for this patient while in the ED?
  Per AAP guidelines, for patients with a simple febrile seizure^2^:
  - No need for lab evaluation only to determine **cause** of a simple febrile seizure (this is something we often do in evaluation of a patient with an unprovoked, afebrile seizure).
  - Lab or imaging workup are generally low yield, and should serve to evaluate the *source* of fever.
  - Neurology consultation, neuroimaging, or EEG not recommended.
  During this ED visit:
  - Targeted physical exam to identify likely source of fever; consider laboratory workup if would be otherwise indicated.
  - In this case, may consider urinalysis and culture if uncircumcised or previous history of urinary tract infection.
  - Consider rapid influenza testing if during season.
  - Give antipyretic.
  - Observe until back to neurologic baseline (usually 1–2 hours).
7. Your patient has returned to his neurologic baseline and has no identifiable source of infection. At this time, you feel he can be safely discharged home. How do you counsel parents regarding:
  - Risk for further seizures?
  - Risk for epilepsy?
  - Need for antiepileptic drug therapy?
  - Fever control at home?
  - Seizure safety?
  - Risk of recurrence of simple febrile seizure
    ○ Age-dependent^16^
      ▪ First episode <12 months: ~50% recurrence
      ▪ First episode >12 months: ~30% recurrence
    ○ Risk of developing epilepsy
      ▪ Single febrile seizure: similar to slightly higher than general population (~1% children).^16^ Current WHO estimates epilepsy prevalence at 0.4%–1%.^17^
      ▪ Recurrent febrile seizures (especially when first event <12 months of age, or with family history of epilepsy): increased risk compared to general population (~2.4%).^18^
      ▪ Complex febrile seizures: increasing risk depending how many complex features (6–50%).^18^
      ▪ Higher risk also if family history of epilepsy
    ○ Other risks^8^
      ▪ Risk of death: No reported cases by consensus of AAP Febrile Seizure Subcommittee
      ▪ Overall long-term outcomes: No adverse long-term health effects from simple febrile seizures
    ○ Need for antiepileptic medications (preventative or intermittent)
      ▪ By recommendation of an AAP subcommittee on febrile seizures, risks of antiepileptic medications outweigh benefits for use in children with only simple febrile seizures
    ○ Fever control
      ▪ Use antipyretics as usual for children with fevers or discomfort during a febrile illness
      ▪ Antipyretic use previously had not been shown to decrease episodes of febrile seizure;^8^ however, a recent randomized controlled clinical trial demonstrated that acetaminophen around the clock reduced repeat episodes of febrile seizure within same febrile illness.^19^
    ○ Seizure safety: What to do during an episode:^20^
      ▪ Place the child on a safe surface (floor or bed) away from hard or sharp things
      ▪ Position them on their side to allow saliva or vomit to drain out of their mouth
      ▪ Don’t put anything in their mouth! They won’t swallow their tongue.
      ▪ Note the time. If a seizure is lasting greater than 5 minutes, call 911.
      ▪ For a briefer episode, phone the primary care physician office or advice line. Often safe in these cases for parents to drive the child in themselves.
▪ Patient Handout: Febrile Seizures from HealthyChildren.org (from the AAP).
8. When do children with febrile seizures potentially need a neurology consultation or referral?
  - Child is not neurodevelopmentally normal^21^
  - Complex febrile seizure— particularly if prolonged or focal seizures

#### Case 2

An otherwise healthy 2-year-old immunized child presents to the emergency department via EMS with a complaint of seizure. Per mother, they estimate that ten minutes prior to arrival, she became unresponsive and began “shaking.” EMS reports they administered 5 mg intramuscular midazolam with no response about 5 minutes ago and have been providing supplemental oxygen. Per mother, the child is fully vaccinated. She has a history of a one-time febrile seizure as an infant and has had no issues since that time. Mother denies any recent travel. She is in daycare and lives at home with her father and 1 older sibling.

On initial exam, the child is having tonic-clonic movements with her head and eyes deviated to the right.

Vitals: Temperature 38.3 degrees Celsius, heart rate 130, blood pressure 80/60, pulse oximetry 93% on nasal cannula at 2 liters/minute

1. What are your initial priorities in management (non-medication management)?
  In the case of pediatric seizure, consider not only “ABC’s,” but also “DEF” for disability, environment/exposure, and fingerstick blood glucose:
  **Airway** - Assess to ensure the patient’s airway is patent with chin lift, jaw thrust
  **Breathing** - Provide 100% oxygen via non-rebreather mask or bag-valve-mask if needed, listen for bilateral breath sounds
  **Circulation** - Check blood pressure and capillary refill
  **Disability** - Do a brief neurologic exam; are pupils equal and reactive? What direction are they deviated? What are the extremities doing?
  **Environment/exposure** - Expose the child to look for any signs of trauma, rashes, etc.
  **Fingerstick blood glucose -** Check a point of care glucose immediately for all patients with seizure.
  Additionally, 2 large-bore IVs should be established immediately, and oxygen should be provided. The patient should be placed on a cardiac monitor (IV, O2, Monitor).
2. The patient’s blood sugar returns at 110 mg/dl. The patient continues to seize. You would like to give another dose of benzodiazepine -- what are your options? Anything else you would like to give at this time? Note: The child is a yellow on Broselow tape, and her mother states she weighs approximately 12 kg.
  List at least 3 options, with appropriate dosages and routes.[Table t5-jetem-5-3-t1]
  The mainstay of initial treatment of pediatric seizures are benzodiazepines. Ideally, intravenous access should be established and either lorazepam or midazolam may be administered. If no intravenous access is immediately available, intramuscular, buccal, or intranasal midazolam may be administered, or rectal diazepam (though generally not used under age two). Appropriate dosage and early onset of seizure medication administration are important![Table t6-jetem-5-3-t1]
  Also, since the patient is febrile, acetaminophen per rectum should be given as well. You may consider a 10–20 ml/kg fluid bolus as well.
3. Despite your initial interventions, the child continues to seize; it has been about 20 minutes since the onset. Your nurse was able to obtain intravenous access. What is your concern now? Does this change your concern? What is your next option for the treatment of this seizure? Include dosing/route of administration.
  One must now consider a complex febrile seizure because it has a prolonged time course (>15 minutes). Other features of complex febrile seizure include: focal seizure, recurrent seizure within 24 hours of onset, or back-to-back seizures without return to neurologic baseline.
  Regarding treatment, after 1–2 doses of an appropriate weight-based administration of intravenous benzodiazepine, a reasonable 2nd line agent in a pediatric child >1 month is fosphenytoin. Dosing is 20 mg/kg for a loading dose, so in a 12 kg child the initial dose is 240 mg.^12^
4. Would you like to obtain any further workup on the patient at this time?
  Up to 25%–30% of seizures are classified as complex febrile seizures.^21^ Laboratory testing may be considered based on clinical judgment and would include testing such as a complete blood count, complete metabolic panel, lactic acid, blood culture, urinalysis with culture, and urine drug screen. An important note is that a complex seizure rarely indicates that a patient has an underlying bacterial meningitis (approximately 0.9% of vaccinated patients in one study).^22^ Therefore, lumbar puncture should be reserved for cases where patients have other risk factors (non-immunized status, age younger than 12 months of age, partial treatment with antibiotics, physical exam signs suggestive of bacterial meningitis).^14^ Additionally, if a patient returns to baseline and is well-appearing following a complex febrile seizure, research has found the yield of neuroimaging to be low and is not generally recommended unless clinical judgment or other signs are concerning for such.^21^
5. Despite your 3rd treatment, the patient continues to seize for an additional 10 minutes. She is becoming more hypoxic, and has a capillary refill of 3 seconds. What intervention(s) would you take at this time?
  Reassess ABC’s and consider fluid bolus of 10–20 ml/kg if not already given. Also consider repeat blood glucose. Consider alternate source of recurrent seizure if history is fitting, such as isoniazid overdose. If the patient is hypoxic and not protecting her airway, you should consider intubation at this time.
  There are a few options for treatment if agents such as fosphenytoin fail; referral to a pediatric intensive care physician as well as pediatric neurologist is indicated. Options include: loading dose of a 2nd antiepileptic drug (AED) or continuous infusion of either phenobarbital or midazolam.
6. After your interventions, the patient’s pupils are now midline, 3 mm and reactive, and the child is not moving. What are your next steps in workup, and the ultimate disposition?
  - You should pursue laboratory testing, lumbar puncture, and neuroimaging, as well as initiate broad-spectrum antibiotics; one may also consider early treatment with acyclovir for coverage for herpes simplex virus. Review of lab work and treatment of any laboratory abnormalities should be corrected.
  - Disposition: Early consultation to pediatric neurology should be performed for initiation of EEG to determine if there is continued seizure activity. The patient should be admitted to the pediatric intensive care unit.

#### Case 3

A 2-week-old newborn female is brought to the emergency room by her parents after having a witnessed generalized seizure at home which resolved after 2 minutes. Per their report, she was born at term via spontaneous vaginal delivery with no complications; mother was otherwise healthy and received full prenatal care. On your initial examination, she is no longer seizing but appears to have poor tone and a weak cry. Patient weighs approximately 3.5 kg.

1. What is your differential diagnosis for this newborn infant? What questions can you ask family and examinations can you perform to develop your prioritized differential diagnosis?[Table t7-jetem-5-3-t1]

Upon questioning, you note that the family has recently become homeless and are struggling financially. The mother states that she has been mixing 1 scoop of infant formula to 6 ounces of water to make the formula last longer until her next Women, Infants, and Children’s (WIC) appointment.

2. What is your concern at this time? What is your initial management and workup?
  - Establish intravenous access
  - Obtain point of care glucose stick, complete blood count with differential, complete metabolic panel, cerebral spinal fluid (CSF) studies, head computed tomography, blood/urine/CSF cultures because the child is 2 weeks and requires a full workup.[Table t8-jetem-5-3-t1]
3. Based on these laboratory studies, what is your suspected diagnosis? How is this defined?
  Based on the laboratory studies, there is concern for hyponatremia secondary to watered down infant formula. Hyponatremia in infants is frequently seen when parents water-down formula in order to make the formula last longer. The appropriate way to mix formula is 1 scoop of formula to 2 ounces of water. Increasing the water content causes an increase in free water and decrease in serum sodium in the patient’s bloodstream. Hyponatremia in children is defined by serum sodium <135mEq/L. It is a disorder of water imbalance. It can be classified as hypovolemic hyponatremia, euvolemic hyponatremia, or hypervolemic hyponatremia.^23^
  - Hypovolemic hyponatremia: Net loss of sodium in excess of net loss of water
    ○ Causes: vomiting, diarrhea, excessive sweating, diuretic therapy, mineralocorticoid deficiency, renal tubular acidosis
  - Euvolemic hyponatremia
    ○ Causes: SIADH^24^ (Syndrome of Inappropriate Antidiuretic Hormone secretion)
  - Hypervolemic hyponatremia: excess of free water
    ○ Causes: heart failure, nephrotic syndrome, cirrhosis
4. The patient suddenly begins seizing again. What is your immediate management (including medication dose and route)?
  - Evaluate airway, breathing, and circulation
  - Considering hyponatremia, patient requires immediate administration of 3–5 ml/kg 3% hypertonic saline. This patient weighs 3.5 kg, so the dose would be approximately 10.5 mg of 3% hypertonic saline intravenously. This can be safely given peripherally
5. After your intervention, the patient’s seizure stops and her vitals are stable. What is your ultimate disposition? What is the feared complication of correcting for this problem too rapidly?

The patient should be admitted to a pediatric intensive care unit for close monitoring of electrolytes and slow correction of hyponatremia. The feared complication of too rapid a correction is osmotic demyelination syndrome (central pontine myelinolysis).^25^ This usually occurs in response to rapid correction of chronic hyponatremia <120mEq/L.

#### Case 4

A 5-year-old boy with a history of congenital hydrocephalus with a VP shunt presents to the emergency department with a reported seizure at home. Per the patient’s mother, he has complained of headache for the last 4 hours and was witnessed to have a generalized seizure lasting three minutes that self-resolved. On exam he appears sleepy, but is moving all extremities and following some commands from his mother. On ocular exam he appears to have dilated pupils with an upward gaze palsy.

Vitals: Temperature 37.8 degrees Celsius, heart rate 120, blood pressure 100/55, respiratory rate 26, oxygen saturation 98% on room air

1. Based on the history above, what is your differential diagnosis for this patient? What is your initial workup and/or management?
  Although shunt failure should be high on the differential, one should consider a broad differential diagnosis for the altered pediatric patient with seizure. Diagnoses should include intracranial hemorrhage, non-accidental trauma, febrile seizure, meningitis/encephalitis, intracranial mass, and toxic ingestion. In terms of laboratory workup, a blood glucose is the most important test to order. One may also consider complete blood count, basic metabolic panel, urinalysis with culture, blood culture, and urine toxicology screen.
  Imaging including a “shunt series” consisting of anteroposterior (AP) and lateral views of the skull, chest and abdomen should be obtained. This will evaluate for potential fracture, disconnection, migration, or misplacement of your shunt system, but gives no insight into the VP shunt function.^26^
  To evaluate ventricular size, providers can obtain either CT or MRI imaging. Both are adequate in evaluating ventricular size. One must weigh the advantages of preventing radiation exposure with CT against that of obtaining rapid imaging available with MRI.^27^
2. Please name each component of a standard VP shunt shown in figure 1. What is the function of the VP shunt? How does it work?
  A)
  B)
  C)[Fig f3-jetem-5-3-t1]
  **Function:**
  - Ventricular Catheter: Catheter usually introduced into the ventricular system by burr hole that then travels outside of the calvarium.
  - Valve Chamber: Subcutaneous chamber that connects to the ventricular catheter. Establishes pressure gradient and ensures drainage away from the ventricle. Sometimes there is a separate reservoir for testing patency, pressure measurement and CSF (Cerebrospinal fluid) sampling.
  - Distal Tubing: Runs subcutaneously from the valve chamber to drainage area which is usually the peritoneal cavity. Uncommonly this can drain to the right atrium, gallbladder, pleura or ureter.^28^
3. On re-evaluation, the patient continues to be drowsy, with no change in clinical status. Your labs return as unremarkable. The CT scan shows acute hydrocephalus; therefore, you are concerned that the shunt may be malfunctioning. What is your next step in management? What are the possible causes of shunt malfunction? List 4 below:
  Next step in management: Contact neurosurgeon for emergent evaluation.
  Possible causes of shunt malfunction:
  1. **Obstruction:** This is the most common cause of shunt malfunction. Common causes of obstruction are tissue debris, clot, infection, kinking or pseudocyst formation. These patients will present with classic signs of increased ICP (increased intracranial pressure) including headache, nausea, vomiting, altered mental status, and cranial nerve palsy.^29^
  2. **Mechanical Failure:** Usually secondary to fracture, disconnection, migration, or misplacement. Given that CSF drainage is usually interrupted, these patients will also present with classic obstruction symptoms.^29^
  3. **Over drainage:** Also known as slit ventricle. The tissues actually occlude the orifices of the proximal shunt. As intracranial pressure increases, the occluding tissue is disengaged, which allows drainage to resume. This phenomenon is cyclical and is responsible for the episodic or waxing and waning increased ICP symptoms.^29^
  4. **Loculation:** Describes a separate, noncommunicating CSF accumulation within a ventricle. The shunt device is not able to drain the entire ventricular system, leaving behind enlarging pockets of fluid that may have compressive sequelae. These patients may present with asymmetric neurologic deficits in the setting of classic increased intracranial pressure symptoms.^30^
  5. **Abdominal Complication:** The most common abdominal complication is pseudocyst formation. These are localized abdominal fluid collections that form around the peritoneal catheter. These are usually secondary to infection and are asymptomatic until they become large enough to cause abdominal pain.^29^
4. The consultant is recommending admission to their service and requesting an MRI. Is it safe for patients with VP shunts to have an MRI?

MRI can be safely obtained in patients with VP shunts and can be diagnostically helpful. Rapid MRI protocols are becoming more common and are recommended if your facility has this capability. However, exposure to strong magnetic fields and some MRI units can change the valve pressure setting, so all patients should have the setting verified after any exposure to strong magnetic fields.^26^
